# Abnormal Regional Spontaneous Neural Activity in Nonarteritic Anterior Ischemic Optic Neuropathy: A Resting-State Functional MRI Study

**DOI:** 10.1155/2020/8826787

**Published:** 2020-09-09

**Authors:** Pengde Guo, Pengbo Zhao, Han Lv, Yan Su, Ming Liu, Yunxiang Chen, Yan Wang, Haiqin Hua, Shaohong Kang

**Affiliations:** ^1^Department of Radiology, Dongfang Hospital, Beijing University of Chinese Medicine, Beijing 100078, China; ^2^Department of Ophthalmology, Dongfang Hospital, Beijing University of Chinese Medicine, Beijing 100078, China; ^3^Department of Radiology, Beijing Friendship Hospital, Capital Medical University, Beijing 100050, China

## Abstract

**Objective:**

To explore altered regional neuronal activity in patients with nonarteritic anterior ischemic optic neuropathy (NAION) and its correlation with clinical performances using the regional homogeneity (ReHo) method, which is based on resting-state functional magnetic resonance imaging (fMRI).

**Method:**

Thirty-one patients with NAION (20 males, 11 females) and 31 age- and sex-matched normal controls (NCs) (20 males, 11 females) were enrolled in the study. All patients underwent ophthalmic examination, including eyesight, intraocular pressure measurement, optimal coherence tomography (OCT), visual field analysis, and fMRI scans. After ReHo was calculated, we investigated group differences in results between the patients and NCs. We analyzed the relationship between ReHo values for different brain regions in patients with NAION and intraocular pressure, visual field analysis, and OCT. A receiver operating characteristic (ROC) curve was used to assess the diagnostic ability of the ReHo method.

**Results:**

Compared with NCs, patients with NAION exhibited higher ReHo values in the left middle frontal gyrus, left middle cingulate gyrus, left superior temporal gyrus, and left inferior parietal lobule. Additionally, they exhibited lower ReHo values in the right lingual gyrus, left putamen/lentiform nucleus, and left superior parietal lobule. ReHo values in the left superior parietal lobule were negatively correlated with right retinal nerve fiber layer values (*r* = −0.462, *P* = 0.01). The area under the ROC curve for each brain region indicated that the ReHo method is a credible means of diagnosing patient with NAION.

**Conclusion:**

NAION was primarily associated with dysfunction in the default mode network, which may reflect its underlying neural mechanisms.

## 1. Introduction

Nonarteritic anterior ischemic optic neuropathy (NAION) typically presents in patients older than 50 years, with an estimated annual incidence of 2.3–10.2 cases per 100,000 people [1, 2] in the United States, 2.9–3.8 cases per 100,000 people in eastern Europe [3], and 6.25 cases per 100,000 people in China [4]. NAION is often characterized by sudden, painless unilateral loss of vision with a characteristic visual field defect and a hyperemic, swollen, or pale optic disc [5]. Risk factors for NAION include hypertension, diabetes, dyslipidemia, anemia, sleep apnea syndrome, and smoking tobacco. Although its detailed pathophysiology is unknown, histopathological studies support the notion that infarction of the short posterior ciliary arteries (vessels that supply the anterior portion of the optic nerve head) is involved [6].

Recently, optical coherence tomography (OCT) has been used to successfully detect NAION. OCT is a noninvasive technology that can detect microstructure changes in retinal layers, which is useful information for diagnosing NAION. Importantly, OCT can reveal changes in the integrity of the intermediate line and thickness of the outer nuclear layer of the retina. However, OCT only focuses on abnormal changes in the eyes. The visual system extends beyond the eye and retina to the optic nerve and the visual cortex of the brain. Whether NAION leads to dysfunction in these parts of the visual system remains unknown.

Based on the blood oxygen level-dependent (BOLD) contrast technique, functional magnetic resonance imaging (fMRI) is an ideal choice for assessing cortical structural/functional abnormalities in NAION because of its high spatial resolution, noninvasiveness, and ability to reflect neuronal activity. A previous study using task-based fMRI found that activation in bilateral occipital cortex was lower in patients with NAION than in healthy controls [7]. In contrast to task-based fMRI studies, resting-state fMRI (rs-fMRI) can reflect background neurophysiological processes and abnormal neuronal activity without external stimulation [8]. Regional homogeneity (ReHo) is a reliable and sensitive method for measuring rs-fMRI that is thought to evaluate the local synchrony of adjacent voxels across the entire brain during resting states [9]. The ReHo method has been successfully used to assess neurological damage in eye diseases such as optic neuritis [10] and retinal detachment [11]. However, spontaneous changes in neural activity remain unclear in patients with NAION. Here, we used ReHo analysis to test our hypothesis that NAION is associated with abnormal neuronal activity in the visual cortex.

## 2. Materials and Methods

### 2.1. Participants

Thirty-one patients with NAION were recruited from the ophthalmology department at Dongfang Hospital affiliated with Beijing University of Chinese Medicine. NAION was diagnosed by an experienced ophthalmologist with 5 years of experience. Among the 31 patients, 16 had unilateral NAION (left eye: 8, right eye: 8) and 15 had bilateral NAION, either sequentially or simultaneously. The following diagnostic criteria were applied: (1) a typical clinical history of sudden, painless, and monocular visual loss or successive bilateral visual loss; (2) received standardized treatment and evaluation in our hospital; and (3) no history of coronary artery disease, hypertension, sleep disorders, or drug addiction. Exclusion criteria included the following: (1) systemic features suggesting optic neuritis, giant cell arteritis, or posterior ischemic optic neuropathy, or a history of optic tumor or other ocular diseases; (2) symptoms of neurological disorders, mental disorders, or the inability or unwillingness to cooperate; and (3) abnormal function in the liver or kidney.

Thirty-one normal controls (NCs) from the university (students) and hospital (staff) were also enrolled in the study. All the NCs were age and sex matched and met the following criteria: (1) no history of ocular disease or symptoms of neurological disease and (2) unaided eyesight > 1.0 on the vision chart.

No participant had any contraindication for MRI scanning, such as claustrophobia or irremovable MRI-incompatible metal in the body. All participants underwent a vision acuity test, intraocular pressure measurement (IOP), visual field (VF) test, OCT to measure retinal nerve fiber layer (RNFL) thickness, and MRI scanning.

The study was approved by the medical research ethics committee and institutional review board of Dongfang Hospital affiliated with Beijing University of Chinese Medicine, Beijing, China. All participants were asked to wear sponge earplugs and a black blinder during MRI scanning. In addition, the methods, potential risks, and purpose of the study were explained to each participant and all provided written informed consent.

### 2.2. Imaging Data Acquisition

MR imaging was performed on a 1.5 Tesla Philips Intera Achieva system (Royal Philips, Amsterdam, and the Netherlands) with an eight-channel head coil. During the scan, participants were asked to keep their eyes open, remain motionless, and not to think about anything during the functional scans.

General sagittal and axial T1-weighted turbo spin-echo (TSE) images, T2-weighted fast field-echo (FFE) images, and short T2-inversion recovery (STIR) images were acquired.

Resting-state fMRI was obtained using an echo planar imaging (EPI) pulse sequence with each scan. Thirty-five axial slices were acquired with the following parameters: repetition time = 3000 ms, echo time = 30 ms, flip angle = 90, field of view = 220 mm × 220 mm, matrix = 64 × 64, thickness = 3.6 mm, and gap = 0.72 mm. Total scan time was 5.06 minutes with 30.36 seconds for each slice. Further, high-resolution structural images (3D BRAVO) were acquired with the following parameters: matrix = 256 × 256, field of view = 256 mm × 256 mm, thickness = 1.0 mm, number of excitation (NEX) = 2, repetition time = 6.5 ms, echo time = 3.2 ms, and flip angle = 8. Each T1 3D-BRAVO contained 161 images.

### 2.3. fMRI Data Analysis

Functional data processing was conducted using DPARSF (Data Processing Assistant for Resting State fMRI) (http://www.rfmri.org/DPARSF_V2_3), which is based on SPM8 (Statistical Parametric Mapping) and REST (Resting-State fMRI Data Analysis Toolkit) (http://restfmri.net/forum/REST_V1.8), and was implemented in MATLAB 2014a (MathWorks, Natick, MA, USA). Preprocessing comprised the following steps: (1) transform EPI DICOM files into NIFTI files, (2) remove first 10 volumes for signal equilibrium and participant adaptation to the scan environment, (3) slice timing correction, (4) head motion correction: to estimate translation and rotation for each participant (any participant whose translation was greater than 1.5 mm maximum shift along any axis (*x*, *y*, or *z*) or whose rotation motion was greater than 1.5° in any direction was dismissed), and (5) fMRI images were spatially normalized and resampled to a standard stereotactic Montreal Neurological Institute (MNI) space using the echo planar-imaging template and coregistered at a resolution of 3 mm × 3 mm × 3 mm.

After preprocessing, the linear trend of the time series was removed and temporally bandpass filtered (0.01–0.08 Hz) to reduce the effect of physiological high-frequency respiration, low-frequency drift, and cardiac noise. Then, individual ReHo maps were generated for each participant based on Kendall's coefficient of concordance (KCC) of the given voxel time series with its nearest 26 neighboring voxels. In addition, we made normalized ReHo maps because the averaged KCC for the whole brain can divide the KCC among each voxel. Finally, the remaining images were smoothed with a Gaussian kernel with a full-width-at-half-maximum of 4 mm × 4 mm × 4 mm.

### 2.4. Statistical Analysis

Independent sample *t*-tests were performed using SPSS 17.0 software (SPSS, Inc., Chicago, IL) to compare clinical data between patients with NAION and NCs. Pearson's linear correlation analyses were used to explore the correlation between the patient ReHo values and the clinical parameters with a statistical significance threshold of *P* < 0.05. The receiver operating characteristic (ROC) curves and the area under the curves (AUC) were used to analyze the ReHo values in the different brain regions, which were compared between the two groups of participants.

The final functional MRI results were presented by xjview toolbox (https://www.alivelearn.net/xjview) and REST software. REST was used for statistical analysis. Two-sample *t*-tests were used to evaluate the differences in ReHo values between patients and NCs with sex, age, and duration of disease as covariates of no interest. Voxels with a *P* < 0.05 (corrected for multiple comparisons using a false discovery rate (FDR) corrected threshold of *P* < 0.05) and cluster size > 23 voxels indicated a significant difference between patients and NCs.

## 3. Results

### 3.1. Demographics and Visual System Measurements

We found no significant differences in age or sex between patients with NAION and the NCs. Compared with the NCs, patients with NAION had significantly worse visual acuity, thinner RNFLs, and lower mean sensitivity (MS) (both left and right, *P* < 0.05). Furthermore, patients had significantly higher IOP and mean deficiency (MD) (both left and right, *P* < 0.05). Details are presented in Table 1.

### 3.2. ReHo Differences

**Figure 1:**
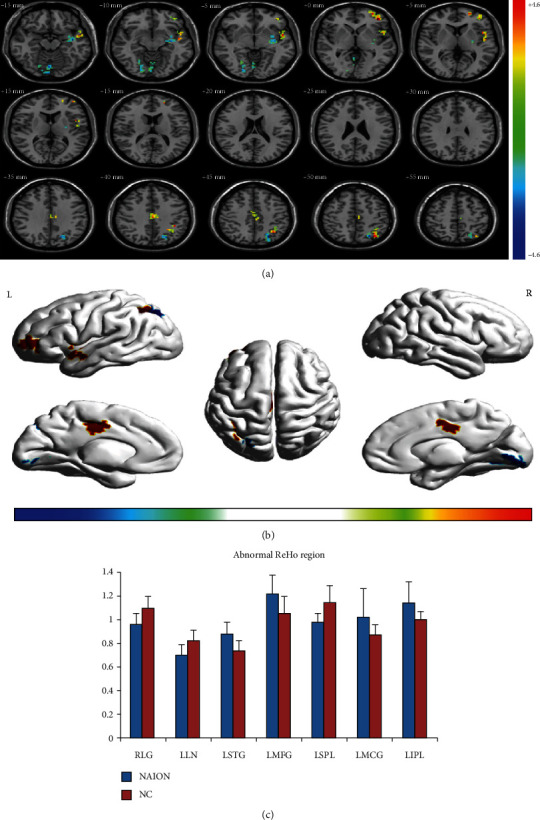
Spontaneous brain activity in patients with NAION and healthy participants Significant differences in activity were observed in patients with NAION in the left middle frontal gyrus, left middle cingulate gyrus, left superior temporal gyrus, left inferior parietal lobule, right lingual gyrus, left putamen/lentiform nucleus, and left superior parietal lobule (false discovery rate corrected, cluster size > 23 voxels, *P* < 0.05) (a, b). The mean ReHo values for NAION and NC groups (c). Abbreviations: NAION: nonarteritic anterior ischemic optic neuropathy; NCs: normal controls; ReHo: regional homogeneity; L: left; R: right.

Compared with NCs, patients with NAION exhibited significantly higher ReHo values in the left middle frontal gyrus, left middle cingulate gyrus, left superior temporal gyrus, and left inferior parietal lobule. They also exhibited lower ReHo values in the right lingual gyrus, left putamen/lentiform nucleus, and left superior parietal lobule. Details are presented in Table 2 and Figure 1.

### 3.3. Correlations between ReHo Values and Clinical Data

**Figure 2:**
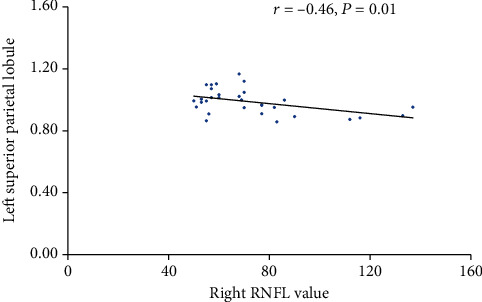
Correlations between right RNFL and the ReHo value of the left superior parietal lobule Abbreviations: REFL: retinal nerve fiber layer thickness; *r*: Pearson's correlation coefficient.

The relationship between ReHo values and disease duration, visual acuity, VF, IOP, and RNFL were examined for each region. We found that thickness of the right RNFL was negatively correlated with the ReHo signal value in the left superior parietal lobule (*r* = −0.462, *P* = 0.01) (Figure 2). We did not find any other correlation between ReHo values and clinical data.

### 3.4. Receiver Operating Characteristic Curve

**Figure 3:**
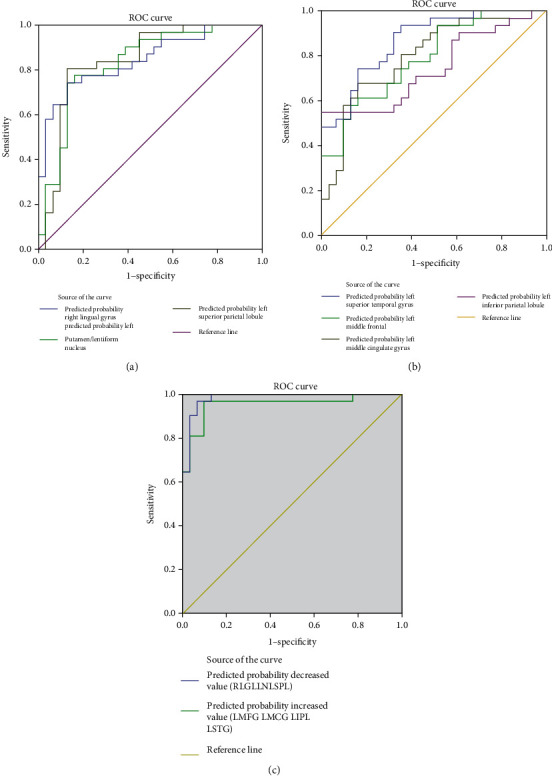
ROC curve analysis of the different ReHo values for abnormal brain regions in patients with NAION. (a) ROC curve for patient regions with high ReHo. The area under the ROC curve was 0.790 for the left middle frontal gyrus (*P* < 0.0001; 95%CI = 0.680 − 0.900), 0.797 for left the middle cingulated gyrus (*P* < 0.0001; 95%CI = 0.685 − 0.909), 0.869 for the left superior temporal gyrus (*P* < 0.0001, 95%CI = 0.783 − 0.954), and 0.745 for the left inferior parietal lobule (*P* < 0.001; 95%CI = 0.620 − 0.870). (b) ROC curve for patient regions with low ReHo. The area under the ROC curve was 0.846 for the right lingual gyrus (*P* < 0.0001; 95%CI = 0.749 − 0.943), 0.843 for the left superior parietal lobule (*P* < 0.0001; 95%CI = 0.739 − 0.947), and 0.832 for the left putamen/lentiform nucleus (*P* < 0.0001; 95%CI = 0.728 − 0.937). (c) ROC curve for combined ReHo regions. The area under the ROC curve was 0.983 for the combined regions of low ReHo (see (a) for the regions) (*P* < 0.0001; 95%CI = 0.958 − 1.009). The area under the ROC curve was 0.954 for the combined regions of high ReHo (see (b) for the regions) (*P* < 0.0001; 95%CI = 0.899 − 1.009).

We speculated that ReHo values may be useful diagnostic markers for NAION. Thus, we performed the ROC curve analysis to determine the mean ReHo values for each brain region that differed between the groups. An area under the curve (AUC) above 0.8 indicates that NAION can be diagnosed accurately. We found individual AUCs for the left middle frontal gyrus (0.790), left middle cingulated gyrus (0.797), left superior temporal gyrus (0.869), left inferior parietal lobule (0.745), right lingual gyrus (0.846), left superior parietal lobule (0.843), and left putamen/lentiform nucleus (0.832). The combined AUC for the regions that exhibited lower ReHo values in patients (left superior parietal lobule, left putamen/lentiform nucleus, and right lingual gyrus) was 0.983. The combined AUC for the regions that exhibited higher ReHo values in patients (left middle frontal gyrus, left middle cingulated gyrus, left superior temporal gyrus, and left inferior parietal lobule) was 0.954 (Figure 3).

## 4. Discussion

During the resting-state condition, the default mode network (DMN) is continuously activated [8]. The DMN contains numerous areas, including the medial frontal cortex, medial temporal lobes, inferior parietal cortex, and anterior/posterior cingulate cortex [8, 12]. Many activities that have an awareness component are related to the DMN, such as anxiety [13], depression [14], and cognition [15]. Previous studies have identified several diseases that lead to DMN dysfunction, such as Parkinson's disease [16], Alzheimer' s disease [15], and multiple sclerosis [17]. Vacchi et al. [17] reported that patients with MS exhibited abnormal DMNs, which were related to poorer cognitive and behavioral outcomes. Shao et al. [10] found that patients with optic neuritis showed low ReHo values in the left middle temporal gyrus, right superior temporal gyrus, left middle frontal gyrus, bilateral anterior cingulate cortex, and bilateral superior frontal gyrus. They also found high ReHo values in the right inferior parietal lobule. Jiang et al. [18] found that patients with primary angle-closure glaucoma showed abnormal activation of areas in the visual cortices, frontal lobe, frontoparietal network, and the DMN. In support of these findings, here, we found that patients with NAION had higher ReHo values in the left middle frontal gyrus, left middle cingulate gyrus, left superior temporal gyrus, and left inferior parietal lobule. At the same time, they had lower ReHo values in the right lingual gyrus, left putamen/lentiform nucleus, and left superior parietal lobule. As an important aspect of rs-fMRI studies, ReHo analysis may provide information that helps us understand more about NAION-related functional reorganization in the brain. The brain is a whole entity rather than a single individual brain area. Dysfunction in one region leads to spontaneous brain activity in other brain regions. Therefore, the lower ReHo values in the left superior parietal lobule indicates that NAION might damage the DMN, while the higher ReHo in the left middle frontal gyrus, left middle cingulated gyrus, left superior temporal gyrus, and left inferior parietal lobule may reflect compensation in the DMN that helps maintain the stability of the internal network.

The lingual gyrus, located in area V2 of the visual cortex, is a key part of the visual cortex that receives feedforward connections from V1. Additionally, V2 plays a critical role in object and shape visual processing [19] and stereo vision [20]. Additionally, the lingual gyrus is thought to be involved in processing visual memory [21] and is the termination of Meyer's loop, which carries visual information from the contralateral superior visual field. Chen et al. [22] observed that patients with primary angle-closure glaucoma showed significantly lower ReHo values in area V2 compared with NCs. Using an optimized voxel-based morphometry, Chen et al. [23] found that primary open-angle glaucoma might lead to significant reduction of gray matter volume in bilateral visual cortex. By analyzing changes in the brain activity, Shao et al. [24] found abnormal ReHo levels in the middle occipital gyrus and the lingual gyrus in patients with strabismus and amblyopia. In addition, Aguirregomozcorta et al. [7] investigated cortical reorganization in 9 patients with NAION using task-based fMRI. They found that occipital activation was lower in patients than in controls when stimulating the affected eye. In the current study, we observed that the ReHo index in the lingual gyrus of patients with NAION was lower than that in the controls, which was consistent with the results from previous studies. The lower ReHo might reflect cognitive impairment in NAION. Thus, we speculated that NIAON might lead to impaired function in V2.

The middle frontal gyrus (MFG), lying between the inferior and superior frontal gyri, has been widely reported to be involved in contingency awareness [25] and cognition [26]. Moreover, the MFG plays a critical role in the parietoprefrontal pathway [27], which are involved in visuospatial working memory [28]. Griffis et al. [29] found that patients with early-onset blindness showed increased functional connectivity between the frontal and occipital lobes. In the present study, therefore, the higher ReHo in the cluster of regions in the left MFG may reflect compensation of visual function and strengthening the parietofrontal networks in NAION. The result suggests that crossmodal plasticity of the parietoprefrontal pathways occurs in individuals with NAION.

The superior temporal gyrus (STG) is the secondary auditory area and plays an important role in auditory processing [30] and auditory memory [31]. It is also associated with visual search insights [32] and visual information processing [33]. The increased ReHo value that we observed for spontaneous brain activity in the STG might reflect plasticity that compensates for NAION-related damage to visual function. This potential compensatory mechanism has also been suggested in participants with other visual deficits, such as neuromyelitis optica [34] and blindness [35], and the mechanism could represent general changes that enable people with impaired vision to perform sensory-guided motor behaviors. Therefore, we speculate that NAION might lead to dysfunction of the auditory and visual information processing.

The inferior parietal lobule (IPL) plays an important role in visual word recognition [36]. Dysfunction of the IPL is also found in some diseases such as Alzheimer's disease [37] and schizophrenia [38]. We demonstrated that patients with NAION showed increased ReHo values in the left IPL, which might reflect compensation for visual dysfunction in NAION.

The superior parietal lobule (SPL) is another part of the visual pathway, which plays a critical role in visuomotor coordination [39]. It is also involved in audio-visual multisensory [40] and language processing [41]. In our study, we found that patients with NAION showed low ReHo values in the SPL, which might be associated with impaired visuomotor function. Furthermore, we observed that the ReHo value in the left superior parietal lobule was negatively correlated with the thickness of the right RNFL. A previous study showed that patients with NAION had significantly thinner globe RNFLs than the controls [42]. Kernstock et al. [43] found that RNFL thickness in patients with NAION decreased rapidly and was below normal values by Month 2 after onset and had further decreased by Month 4. This progressive thinning between Months 2 and 4 suggests ongoing atrophy of nerve fibers. Similarly, Resch et al. [42] found that compared with the controls, RNFL thickness decreased significantly during the 3 months following disease onset. Moreover, they found that the inner plexiform layer of ganglion cells became thinner throughout the course of the disease [44]. To some extent, the degree to which RNFL thickness is reduced indicates the severity of NAION. Overall, these findings may suggest that when atrophic RNFL damage is severe, it can cause dysfunction of visuomotor coordination in the left SPL.

An initially unexpected—and very interesting—finding was that locations with increased ReHo values were all located in the left hemisphere. Although this could be a coincidence, it might be related to the very important roles that the left cerebral hemisphere plays in visual-word recognition, which have been suggested by neuroimaging and neuropsychological studies [45, 46]. Indeed, studies have demonstrated that visually processing word is more effective in the left cerebral hemisphere than in the right [47, 48]. Therefore, the increased ReHo values that we found in the left cerebral hemisphere might reflect functional reorganization that compensated for impaired visual function that resulted from NAION.

In the present study, ROC analysis was applied to determine the reliability of using ReHo values to diagnose patients. Several neuroimaging studies focusing on ocular diseases have successfully applied ROC analyses to discriminate those with ocular disease from NCs [11, 22, 24, 49, 50]. According to these studies, the ability to discriminate conditions are considered excellent, moderate, fair, and failed when AUC values are 0.9–1, 0.7–09, 0.5–0.7, and less than 0.5, respectively [22, 24, 50]. The present study found that the brain regions with abnormal ReHo values consistently showed a high degree of sensitivity and specificity with higher AUC values. The AUC values for each of these regions were over 0.7. Furthermore, the AUC values for the combined brain regions (higher or lower values than controls) were both over 0.9. Therefore, the results the present study indicate that these abnormal ReHo values (i.e., changes in the brain) can serve as biomarkers for diagnosing NAION.

The current study has several limitations that should be considered. First, NAION typically occurs unilaterally. Involvement of the other eye can occur years after the first eye is affected. However, patients typically visit different clinics because treatment is ineffective. When patients arrive at our hospital, some already exhibit NAION in both eyes. Thus, it was difficult to recruit patients with only one affected eye. Second, patients in the present study did not undertake neuropsychological tests though a few people complained insomnia or irritability. Further research is required to examine this issue in more detail. This limitation could be resolved using larger patient samples.

## 5. Conclusions

The present study revealed that patients with NAION exhibit an abnormal spontaneous brain activity, including a negative correlation with contralateral RNFL. The abnormal spontaneous activity demonstrated that patients with NAION had undergone neural remodeling. The findings may be related to functional brain networks and the DMN, as well as to visual compensation. The current results provide important information that improves our understanding of the inherent neural mechanisms underlying NAION.

## Figures and Tables

**Table 1:** Participant characteristics.

Characteristics	NAION (*n* = 31)	NCs (*n* = 31)	*t* value	*P* value
Age (years)	35~79 (52.74 ± 11.29)	33~66 (50.97 ± 8.20)	0.71	0.482
Sex, male/female	31, 20/11	31, 20/11	NA	NA
Disease duration (years)	6.00 ± 1.12	NA	NA	NA
Vision-right	0.53 ± 0.40	1.08 ± 0.17	-7.14	<0.001
Vision-left	0.57 ± 0.40	1.10 ± 0.17	-6.75	<0.001
IOP-right	14.61 ± 2.38	13.10 ± 1.27	3.16	0.002
IOP-left	15.35 ± 2.03	13.68 ± 1.62	3.60	0.001
RNFL-right (*μ*m)	73.00 ± 23.25	97.58 ± 8.24	-5.55	<0.001
RNFL-left (*μ*m)	79.03 ± 28.51	97.80 ± 6.91	-3.56	0.001
CVF				
MS-right	13.83 ± 9.23	26.96 ± 1.42	-7.83	<0.001
MS-left	16.79 ± 9.04	26.89 ± 1.29	-6.16	<0.001
MD-right	12.76 ± 9.30	0.86 ± 1.33	7.05	<0.001
MD-left	10.47 ± 9.05	0.92 ± 1.28	5.82	<0.001

Abbreviations: SD: standard deviation; NA: not applicable; IOP: intraocular pressure; RNFL: retinal nerve fiber layer thickness; CVF: central vision field; MS: mean sensitivity; MD: mean defect.

**Table 2:** ReHo values for patients with NAION and healthy controls.

Conditions	Brain region	R/L	Peak MNI (mm)			Peak *T* value	Cluster size (mm^3^)
			*x*	*y*	*z*		
NAION > NCs	Middle frontal gyrus	L	-39	42	0	3.6565	93
NAION > NCs	Middle cingulate gyrus	L	0	-21	39	3.1801	65
NAION > NCs	Inferior parietal lobule	L	-33	-69	51	3.1296	66
NAION > NCs	Superior temporal gyrus	L	-51	3	-6	3.1149	88
NAION < NCs	Lingual gyrus	R	21	-87	-6	-2.9064	71
NAION < NCs	Putamen/lentiform nucleus	L	-39	-9	-15	-3.5122	95
NAION < NCs	Superior parietal lobule	L	-24	-69	48	-4.4565	82

Note: two-sample *t*-tests were used test for differences between the NAION and NC groups. The threshold was set with *P* < 0.05, corrected for multiple comparisons using false discovery rate. Abbreviations: NAION: nonarteritic anterior ischemic optic neuropathy; NCs: normal controls; ReHo: regional homogeneity; L: left; R: right; MNI: Montreal Neurological Institute.

## Data Availability

The datasets generated and analyzed during the current study are available from the corresponding author on reasonable request.
